# *Strongyloides stercoralis* coinfection impairs immune control of *Mycobacterium tuberculosis* in TB infected individuals

**DOI:** 10.3389/fimmu.2025.1735029

**Published:** 2026-01-09

**Authors:** Anuradha Rajamanickam, Bindu Dasan, Arul Nancy Pandiarajan, Saravanan Munisankar, Sivakumar Shanmugam, Subash Babu

**Affiliations:** 1 National Institute of Health-National Institute of Allergy and Infectious Diseases-International Center for Excellence in Research, Chennai, India; 2 Department of Bacteriology, Indian Council of Medical Research (ICMR)-National Institute for Research in Tuberculosis, Chennai, India; 3 Laboratory of Parasitic Diseases, National Institute of Allergy and Infectious Diseases, National Institutes of Health, Bethesda, MD, United States

**Keywords:** cytokines, immune modulation, MGIA, *Strongyloides stercoralis* infection, Th1/Th2 balance, tuberculosis infection

## Abstract

**Background:**

Helminth co-infection are common in tuberculosis (TB) endemic regions and alter host immunity. However, their impact on immune responses in TB infection (TBI) remains incompletely defined.

**Methods:**

We analyzed QuantiFERON (QFT) supernatants and peripheral blood mononuclear cells (PBMCs) from TBI+Hel+ (*S. stercoralis(Ss)* infection) (n=15) and TBI+Hel− (n=23) individuals. Cytokine levels were assessed at baseline and following TB antigenic stimulation (TBAg1 and TBAg2), as well as mitogenic stimulation. PBMCs were stimulated with *Mycobacterium tuberculosis* (*M. tuberculosis*) H37Rv, and antimycobacterial immunity was assessed using an *in vitro* mycobacterial growth inhibition assay (MGIA) and multiplex cytokine assays.

**Results:**

We measured Type 1 (IFN-γ, TNF-α, IL-2), Type 17 (IL-17, IL-22), and proinflammatory cytokines (IL-1α, IL-1β) in QuantiFERON (QFT) supernatants from TBI individuals with (TBI+Hel+) and without (TBI+Hel−) infection. TBI+Hel+ individuals exhibited significantly reduced mycobacterial growth inhibition compared with TBI+Hel− individuals, reflected by higher normalized mycobacterial growth (Δ log10 CFU). Cytokine analysis showed suppression of Th1/Th17 (IFN-γ, IL-2, TNF-α, IL-17, GM-CSF) and proinflammatory cytokines (IL-1α, IL-1β, IL-6, IL-12p70, IL-18) in TBI+Hel+ individuals, alongside increased Th2/regulatory cytokines (IL-4, IL-5, IL-10, IL-13).

**Conclusions:**

*Ss* co-infection impairs host antimycobacterial control by skewing TBI immune responses toward Th2/regulatory pathways at the expense of protective Th1/Th17 mechanisms. Our data demonstrate that the importance of considering *Ss* co-infection into account when developing TB control and vaccination strategies in endemic areas.

## 1 Introduction

Helminth infections affect approximately 1.5 billion people worldwide, predominantly in tropical regions, and typically induce a T-helper 2 (Th2) immune response characterized by IL-4, IL-5, and IL-13 (1). TB infection (TBI), the asymptomatic phase of *Mycobacterium tuberculosis* (*M. tuberculosis*) infection, presents a significant public health challenge due to its potential for reactivation, especially in immunocompromised individuals (2). Approximately 25% of the global population has TBI, and an estimated 5% progress to active TB within two years of infection (https://www.who.int/teams/global-programme-on-tuberculosis-and-lung-health/tb-reports/global-tuberculosis-report-2024^3^). A Th1-mediated immune response, involving cytokines such as IFN-γ and TNF-α, is critical for controlling *Mtb* (2).

Helminths may shift immune responses to *M. tuberculosis*, favoring Th2/regulatory pathways and potentially impairing Th1-mediated control (4, 5). Conversely, some studies suggest helminths might reduce inflammation and tissue damage during active TB, potentially offering protective effects (5, 6). However, the exact mechanisms through which helminths impact TB progression remain unclear.

The Mycobacteria Growth Inhibition Assay (MGIA) is a key tool for evaluating immune responses to *M.tb*, providing insights into the Th1/Th2 balance and how helminth infections modify this response (7). Cytokine profiles from MGIA assays show that *M.tb* typically induces a Th1 response, while helminths promote a Th2 environment, potentially hindering the immune mechanisms necessary for effective *M.tb* control. This shift could lead to increased susceptibility to TB reactivation (2, 4, 8).

Understanding how helminths modulate immune responses in TBI is critical for predicting the outcome of co-infection. Helminth-induced regulatory cytokines, such as IL-10, may suppress Th1 responses, impairing the host’s ability to contain *Mtb* (9). However, some studies also suggest that helminths could reduce immune hyperactivation, potentially minimizing tissue damage in TB (10). Recent studies further reveals that helminth co-infection modulates chemokine and cytokine responses in TBI, with implications for disease progression (5, 11).

Our study aims to investigate cytokine profiles from MGIA assays in individuals with TBI and *Ss* co-infection. In our study population, all helminth infections identified were caused by *Strongyloides stercoralis*; therefore, results refer specifically to *S. stercoralis* co-infection. The goal is to explore how *Ss* induced immune modulation affects the Th1/Th2 balance and *M.tb* growth inhibition, providing insights that could enhance TB management strategies in endemic regions.

## 2 Materials and methods

### 2.1 Ethical statement

Ethical approval was obtained from the Institutional Review Boards of the National Institute of Allergy and Infectious Diseases (NIAID), USA, and the National Institute for Research in Tuberculosis (NIRT), India (NCT04813328, NCT04526613, NIRT-IEC 2020–033 and NIRT-IEC 2020 005). Written informed consent was obtained from all study participants.

### 2.2 Study population

We enrolled 38 individuals with TB infection (TBI) from Tiruvallur and Kanchipuram Districts, Tamil Nadu, South India, between September 2022 and January 2023: 15 with *Ss* co-infection (TBI+Hel+) and 23 without *Ss* co-infection (TBI+Hel−). Participants were aged 22–63 years. These 38 individuals were prospectively enrolled specifically for this study and were not selected from a larger pre-existing cohort. TBI diagnosis was based on positive QuantiFERON-TB Gold Plus (QFT-Plus) results (QIAGEN), absence of pulmonary symptoms, and normal chest radiographs. None of the participants were known contacts of active TB cases. Pregnant or lactating women were excluded from the study. All participants were HIV-negative and anti-TB treatment–naïve. TBI was identified for study enrolment; TBI-positive individuals were referred to TB clinics per public health protocols.

### 2.3 QuantiFERON-TB gold in-tube assay

QFT-Plus testing followed manufacturer guidelines. Heparinized whole blood was incubated at 37°C for 18 hours in TB1, TB2, mitogen, and Nil tubes. In the QFT-Plus assay (Qiagen), the TB1 tube contains long ESAT-6 and CFP-10 peptides that predominantly stimulate CD4^+^ T cell responses, whereas the TB2 tube contains additional short ESAT-6 and CFP-10 peptides designed to activate both CD4^+^ and CD8^+^ T-cell subsets. Tubes were centrifuged at 3,000 g for 15 minutes, and supernatants were collected for IFN-γ measurement (IU/mL). Remaining supernatants were stored at −80°C.

### 2.4 Parasitological examination and anthelmintic treatment

*Strongyloides stercoralis (Ss*) infection was diagnosed by IgG antibodies to the recombinant NIE antigen (12, 13). A single stool sample was examined by Kato-Katz; stool microscopy was negative for other intestinal helminths. *Ss* infection was further confirmed by nutrient agar plate culture (14). Filarial infection was excluded by negative circulating filarial antigen tests. *S. stercoralis* was the only helminth species detected in this study population. All *Ss*-positive participants received ivermectin (12 mg, single dose) and albendazole (400 mg, single dose). Follow-up blood draws and repeat parasitological examinations were performed six months later to confirm clearance.

### 2.5 Mycobacterial growth inhibition assay

*M.tb* H37Rv was cultured in a Middlebrook 7H9 liquid medium (Sigma-Aldrich). The mycobacterial inoculation volume for each batch was titrated to achieve a time to positivity (TTP) of 7 days in MGITs in a BACTEC MGIT 960 machine. The inoculum was titrated to achieve a 7-day time to positivity (TTP) in MGITs; inocula ranged between approximately 10^2–10^3 CFU/mL.

### 2.6 Isolation of PBMCs and mycobacterial growth inhibition assay

Human peripheral blood mononuclear cells were isolated from peripheral blood and resuspended at an approximate concentration of 2–3 × 10^6^ cells per mL of RPMI (containing 10% fetal bovine serum and 2 mM l-glutamine) and benzonase was added to a final concentration of 25 U/mL. Cells were rested at 37°C for 2 hours with 5% CO_2_. Six hundred microliters of RPMI (containing 2 mM L-glutamine and 25 mM HEPES) seeded with 1×10^6^ PBMC and *M.tb* H37Rv was added to duplicate 2-mL screw-cap tubes. The co-cultures were incubated on a 360° rotator at 37 °C for 96 hours, after which tubes were micro-centrifuged at 15,300 *g* for 10 minutes, and the supernatant was carefully removed by pipetting. Cells were lysed with the addition of 500 µL sterile water and the tubes pulse-vortexed at 0, 5 and 10 minutes. Five hundred microliters of lysate were then transferred directly to a BACTEC MGIT tube supplemented with PANTA antibiotics (polymyxin B, amphotericin B, nalidixic acid, trimethoprim, and azlocillin) and OADC enrichment broth. On day 0, duplicate direct-to-MGIT inoculum control tubes were prepared with the same *M. tuberculosis* input as samples. In the end, MGIT tubes were placed on the BACTEC 960 machine and incubated at 37 °C until the detection of positivity by fluorescence. TTP was converted to log10 CFU using standard curves generated by inoculating MGIT tubes with 10-fold mycobacterial dilutions and plating aliquots on 7H11 agar. Linear regression (GraphPad Prism v10) provided equations for TTP-to-CFU conversion. Normalized mycobacterial growth was defined as log10 CFU (sample) − log10 CFU (growth control). The difference between the medians of respective groups is described as Δ X log and was calculated by subtracting the median of the test group from the median of the control group.

### 2.7 Luminex assays for cytokines

Cytokine levels in culture supernatants were measured using a Human Magnetic Luminex Assay (R&D Systems) on the Bio-Rad MAGPIX platform (xPONENT 4.2; Bio-Plex Manager 6.1). Analytes included Interferon gamma (IFNγ), interleukin-2 (IL-2), Tumor Necrosis Factor alpha (TNFα), IL-17, granulocyte-macrophage colony-stimulating factor (GM-CSF), IL-1α, IL-1β, IL-6, IL-12p70, IL-18, IL-4, IL-5, IL-10, and IL-13. The lowest detection limits for cytokines were as follows: GM-CSF, 18.4 pg/mL; IFN-γ, 5.7 pg/mL; IL-1α/IL-1F1, 10.6 pg/mL; IL-1β/IL-1F2, 3.5 pg/mL; IL-2, 3.6 pg/mL; IL-4, 1.1 pg/mL; IL-5, 6.2 pg/mL; IL-6, 9.0 pg/mL; IL-10, 32.2 pg/mL; IL-12p70, 18.5 pg/mL; IL-13, 31.8 pg/mL; IL-17/IL-17A, 9 pg/mL; IL-18/IL-1F4, 2.5 pg/mL; and TNF-α, 12.4 pg/mL.

### 2.8 Statistical analysis

Data were summarized as medians with interquartile ranges (IQR). Between-group comparisons used the Mann–Whitney U test with Holm–Bonferroni correction for multiple comparisons. Correlations were assessed using Spearman’s rank correlation. *Ss*-specific IgG (OD values) and all cytokines measured in QFT or PBMC supernatants were entered as independent variables in a multivariate linear regression model to assess their combined association with MGIA (Δ log10 CFU). Analyses were performed in GraphPad Prism v10.0; significance was set at p ≤ 0.05.

## 3 Results

### 3.1 Study population characteristics

The demographic, hematological, and biochemical characteristics of the study participants are summarized in **Table 1**. There were no statistically significant differences in age, gender, or body mass index (BMI) between the Tuberculosis infected (TBI) individuals with helminth infection (TBI+ Hel+) and those without helminth infection (TBI+). As only *Strongyloides stercoralis* infection was identified among helminth-positive participants, the comparisons presented here specifically reflect the impact of *S. stercoralis* co-infection on immune responses in TBI. However, notable differences were observed in certain hematological parameters. TBI+ Hel+ individuals exhibited significantly decreased levels of hemoglobin (TBI+ Hel+ 11.25 Vs TBI + 13.10 g/dl, p=0.022), Red Blood Cells (RBC) (TBI+ Hel+ 4.75 Vs TBI + 4.05 (10^3^/ml), p=0.034) and lymphocytes compared to TBI+ individuals. In contrast, eosinophil levels (TBI+ Hel+ 0.75 Vs TBI + 0.44 (10^3^/ml), p=0.028 were significantly increased in TBI+ Hel+ individuals compared to TBI+ individuals.

**Table 1:** Demographic and hematological parameters of the study population.

Parameter	TBI+Hel+ (*n* = 15)	TBI (*n* = 23)	*p*
Gender (Male/Female)	7/8	12/11	NS
Median age (range) in Years	44 (29-53)	40 (24-56)	NS
BMI kg/m² GM (range)	24.6 (17.3-30.1)	25.3 (21-32)	NS
Hb gm/dl GM (range)	11.25 (4.8–16.0)	13.10 (5.5–18.8)	0.022
RBC 10^6^/ul GM (range)	4.05 (2.3–5.6)	4.75 (3.8–6.20)	0.033
WBC 10^3^/ul GM (range)	9.05 (6.0 –16.5)	8.72 (5.7 –13.4)	NS
HCT % GM (range)	38.2 (21.0 –52.5)	34.0 (15.2 – 46.8)	NS
PLT 10^3^/ul GM (range)	268.5 (150–420)	254.0 (200–365)	NS
Neutrophil 10^3^/ml GM (range)	5.4 (3.6–7.3)	5.64 (4.3–6.9)	NS
Lymphocyte 10^3^/ml GM (range)	2.40 (1.30–3.55)	3.20 (2.15–4.32)	0.018
Monocyte 10^3^/ml GM (range)	0.76 (0.41–1.16)	0.81 (0.47–1.10)	NS
Eosinophil 10^3^/ml GM (range)	0.82 (0.15–3.54)	0.44 (0.18–1.05)	0.028
Basophil 10^3^/ml GM (range)	0.11 (0.03–0.34)	0.09 (0.05–0.31)	NS
*Ss*-specific IgG (OD units), median (IQR)	1.84 (1.50–2.33)	0.14 (0.08–0.21)	<0.0001

Hb, hemoglobin; RBC, red blood cell; WBC, white blood cell; HCT, hematocrit test; PLT, platelet count; NS, not significant.

### 3.2 Decreased Th1, Th17, and proinflammatory cytokine responses in *Ss* co-infected TBI individuals

**Figure 1:**
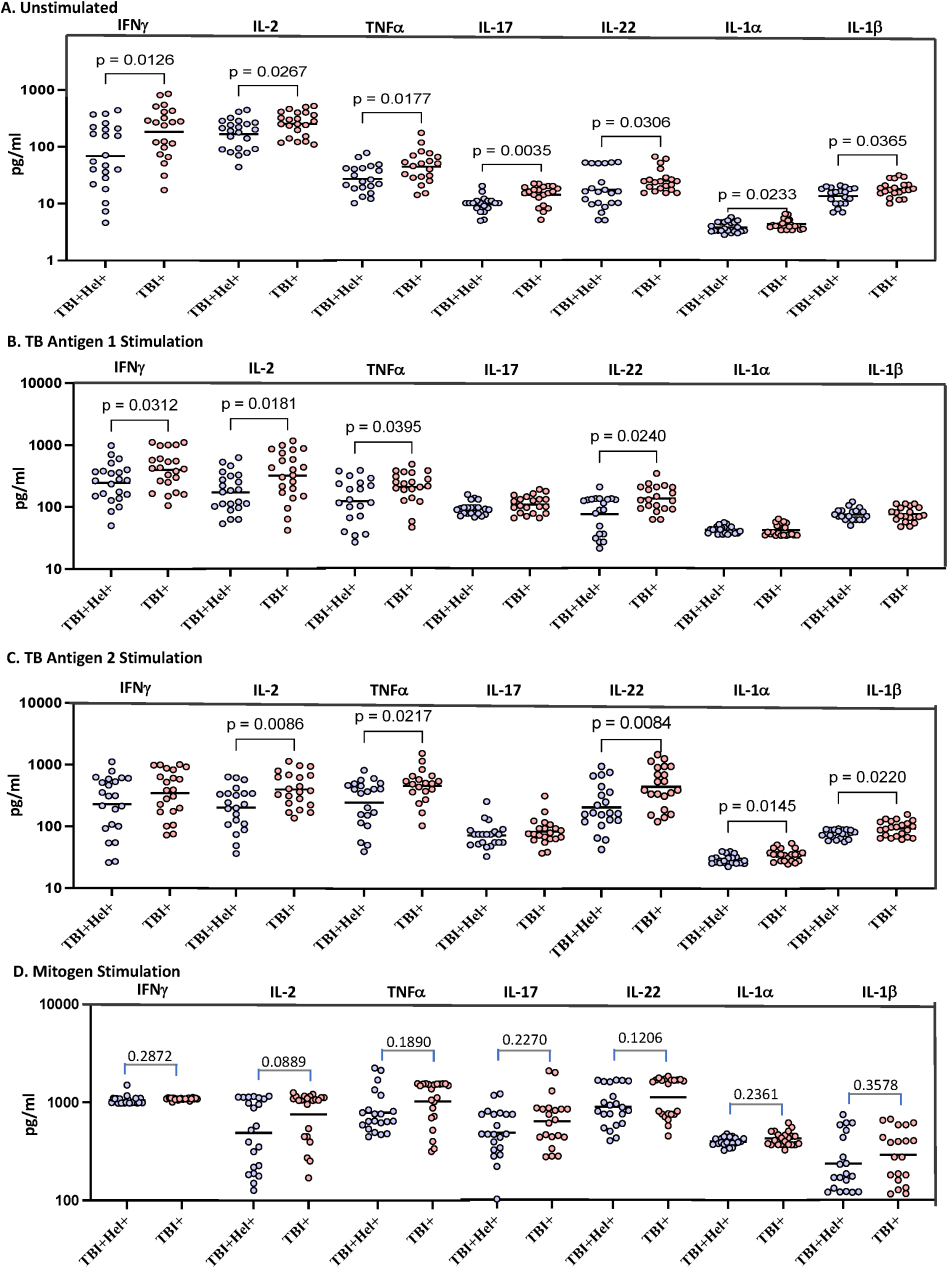
Decreased Th1, Th17, and proinflammatory cytokine responses in *Ss* co-infected TBI individuals.Helminth infection is associated with diminished systemic levels of type 1 and type 17 cytokines TBI. Panels **(A–D)** show QFT supernatant cytokine levels at baseline (Nil), TB antigen 1 (TB1), TB antigen 2 (TB2), and mitogen stimulation in TBI+Hel+ (n=15) and TBI+Hel− (n=23) individuals. Data are displayed as medians with IQR; p values from Mann–Whitney U with Holm–Bonferroni correction.

To determine the influence of coexistent *Ss* infection on type 1, type 17, and proinflammatory cytokines in TBI, we measured the QFT supernatants levels of IFN-γ, TNF-α, IL-2, IL-17, IL-22, IL-1α and IL-1β in TBI+Hel+ and TBI+Hel- individuals. As shown in **Figure 1**, TBI+Hel+ co-infected individuals exhibited significantly decreased levels of Type 1, median (IFN-γ: TBI+Hel+, 63 pg/mL Vs TBI+, 268.1 pg/mL, p=0.0126, IL-2, TBI+Hel+, 195 pg/mL Vs TBI+, 265 pg/mL, p=0.0267; TNF-α: TBI+Hel+, 25.06 pg/mL Vs TBI+, 47.50 pg/mL, p=0.0177; Type 17 (IL-17, TBI+Hel+, 10 pg/mL Vs TBI+, 16 pg/mL, p=0.0035; IL-22, TBI+Hel+, 15.88 pg/mL Vs TBI+, 23.56 pg/mL, p=0.0306 and proinflammatory cytokines, IL1α, TBI+Hel+, 3.45 pg/mL Vs TBI+, 4.07 pg/mL, p=0.0233 and IL1β, TBI+Hel+, 15.74 pg/mL Vs TBI+, 17.42 pg/mL, p=0.0365 at baseline and upon TBAg1 stimulation, median (IFN-γ: TBI+Hel+, 267.5 pg/mL Vs TBI+, 415.1 pg/mL, p=0.0312, IL-2, TBI+Hel+, 175 pg/mL Vs TBI+, 352.9 pg/mL, p=0.0181; TNF-α: TBI+Hel+, 27.38 pg/mL Vs TBI+, 61.11 pg/mL, p=0.0395; and IL-22, TBI+Hel+, 98 pg/mL Vs TBI+, 124 pg/mL, p=0.0240; upon TBAg2 antigenic stimulation median (IL-2, TBI+Hel+, 230 pg/mL Vs TBI+, 381.2 pg/mL, p=0.0086; TNF-α: TBI+Hel+, 40.99 pg/mL Vs TBI+, 68 pg/mL, p=0.0217; Type 17 (IL-22, TBI+Hel+, 28.20 pg/mL Vs TBI+, 78.20 pg/mL, p=0.0084; proinflammatory cytokines, IL1α, TBI+Hel+, 4 pg/mL Vs TBI+, 5.5 pg/mL, p=0.0145 and IL1β, TBI+Hel+, 15.48 pg/mL Vs TBI+, 19.80 pg/mL, p=0.0220 in comparison with TBI individuals alone. In contrast, the upon mitogenic stimulation there was no significant difference between the two groups. Thus, coexistent *Ss* infection is associated with diminished systemic levels of type 1 type 17 cytokines and proinflammatory cytokines in TBI alone individuals.

### 3.3 Decreased mycobacterial growth inhibition in *Ss* infected LTBI individuals

**Figure 2:**
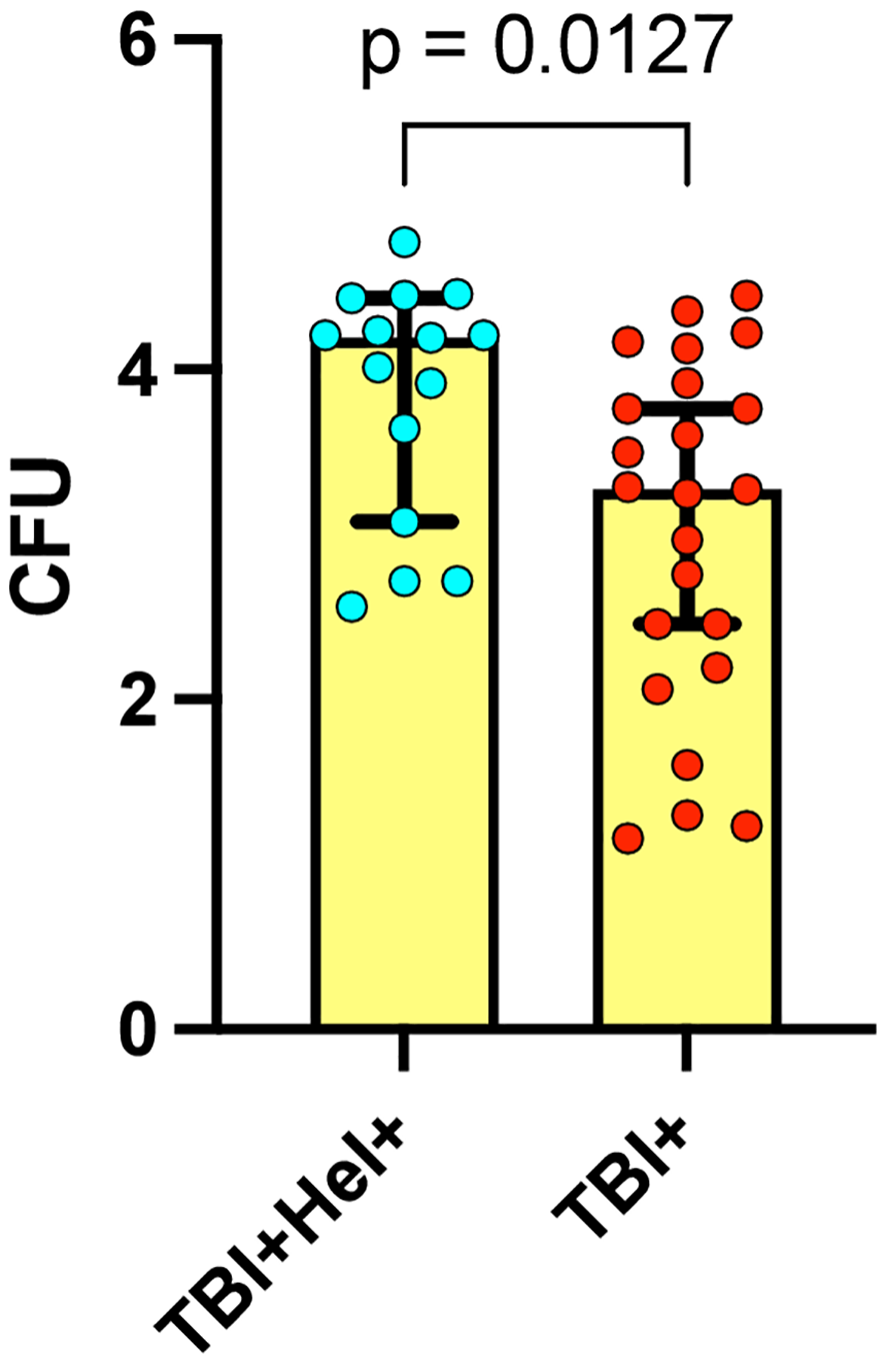
Mycobacterial growth inhibition assay in tuberculosis infected individuals with *Ss* infection. The figure displays MGIA normalized mycobacterial growth (Δ log10 CFU) in TBI+Hel+ and TBI+Hel− individuals. Each point represents one participant; bars show medians. Mann–Whitney U test with Holm–Bonferroni correction was used for all pairwise comparisons.

We assessed the *in vitro* growth inhibition of *Mycobacterium tuberculosis* H37Rv in peripheral blood mononuclear cells (PBMCs) from individuals with latent tuberculosis infection (TBI) with and without *Ss* co-infection. As shown in **Figure 2**, TBI+Hel+ individuals exhibited significantly higher normalized mycobacterial growth (Δ log10 CFU 3.8) than TBI+Hel− individuals (Δ log10 CFU 2.8; p=0.0127), indicating reduced capacity to inhibit *M. tuberculosis* growth in the *Ss* co-infected group.

### 3.4 *Ss* co-infection suppresses both adaptive and innate cytokine responses to *Mycobacterium tuberculosis*

**Figure 3:**
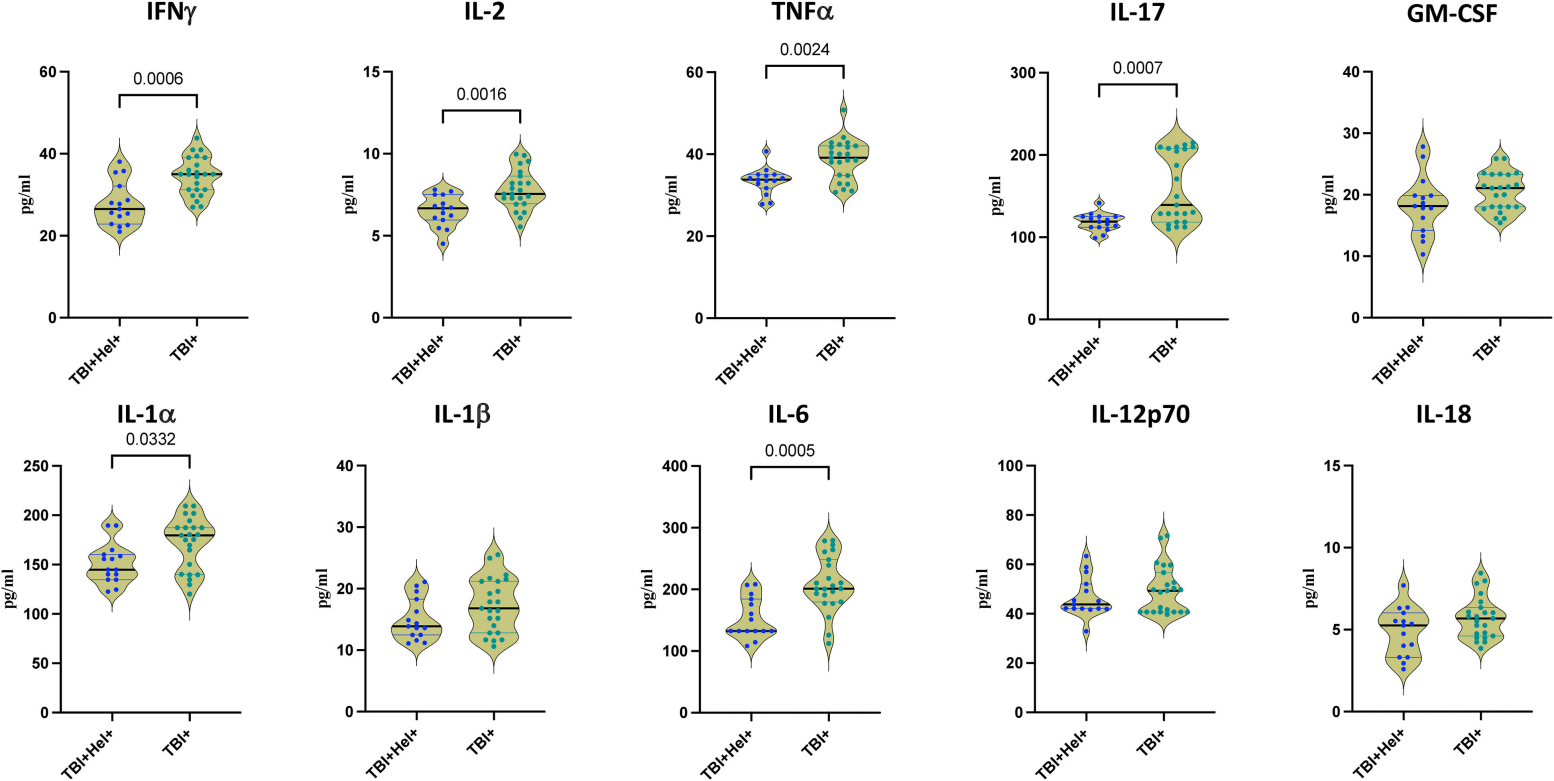
Cytokine secretion profile in tuberculosis infected individuals with *Ss* infection. The figure illustrates the Type 1 and pro-inflammatory cytokine secretion profile in response to *M. tuberculosis* H37Rv in TBI+Hel+ and TBI+Hel- individuals. Cytokines including Interferon gamma (IFNγ), Interleukin-2 (IL-2), Tumor necrosis factor alpha (TNF-α), Interleukin-17 (IL-17), Granulocyte macrophage colony-stimulating factor (GM-CSF), Interleukin-1 alpha (IL-1α), Interleukin-1 beta (IL-1β), Interleukin-6 (IL-6), Interleukin-12 (IL-12p70), Interleukin-18 (IL-18), were measured in the culture supernatant. Each data point represents an individual subject, with the bar indicating the geometric mean (GM) cytokine level. Statistical analysis was performed using the Mann–Whitney U-test with Holm–Bonferroni correction was used for all pairwise comparisons.

We evaluated cytokine responses following stimulation of PBMCs with *Mycobacterium tuberculosis* H37Rv to assess the impact of *Ss* co-infection in individuals with tuberculosis infection (TBI). As shown in **Figure 3**, TBI-helminth co-infected individuals exhibited significantly diminished secretion of Th1 cytokines, median concentrations were IFN-γ (35 pg/mL Vs 26.51 pg/mL), IL-2 (7.6 pg/mL Vs 6.8 pg/mL), and TNF-α (39.1 pg/mL Vs 33.8 pg/mL), compared to TBI+ individuals alone. Furthermore, median concentrations were IL-17 (139.2 pg/mL Vs 119.0 pg/mL) and GM-CSF (21.08 pg/mL Vs 18.2 pg/mL) —key cytokines involved in Th17-mediated immunity and granuloma integrity—were also significantly reduced in the helminth-infected group.

In addition to the suppression of adaptive immune responses, innate inflammatory cytokine production was also markedly impaired. Notably, levels of median concentration were IL-1α (179.7 pg/mL Vs 144.9 pg/mL) and IL-6 (201.2 pg/mL Vs 132.3 pg/mL), were significantly decreased in LTBI-helminth co-infected individuals following *M. tuberculosis* stimulation. Whereas IL-1β (16.8 pg/mL Vs 13.88 pg/mL), IL-12p70 (49.2 pg/mL Vs 43.8 pg/mL) and IL-18 (5.7 pg/mL Vs 5.3 pg/mL) levels were decreased in TBI- *Ss* co-infected individuals but did not show any significant difference when compared with TBI+ individuals alone. These cytokines are critical for early-phase immune activation and effective initiation of host defense mechanisms against TB. Together, these results indicate that *Ss* co-infection leads to broad immune suppression encompassing both innate and adaptive arms of the immune response to *M. tuberculosis*.

### 3.5 Enhanced type 2 and regulatory cytokine responses

**Figure 4:**
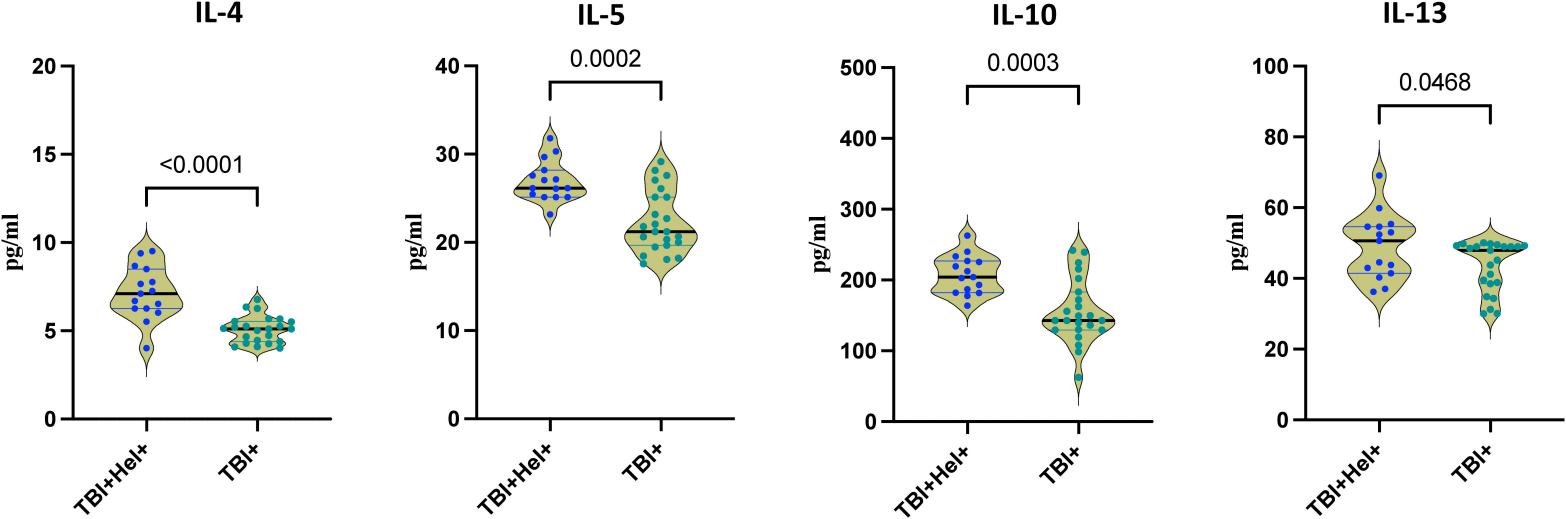
Type 2 cytokine secretion profile in tuberculosis infected individuals with *Ss* infection. The figure illustrates the Type 2 cytokine secretion profile in response to *M. tuberculosis* H37Rv in TBI+Hel+ and TBI+Hel- individuals. Interleukin-4 (IL-4), Interleukin-5 (IL-5), Interleukin-10 (IL-10), and Interleukin-13 (IL-13) were measured in the culture supernatant. Each data point represents an individual subject, with the bar indicating the geometric mean (GM) cytokine level. Statistical analysis was performed using the Mann–Whitney U-test with Holm–Bonferroni correction was used for all pairwise comparisons.

Next, we evaluated the Th2 and regulatory cytokines following stimulation of PBMCs with *Mycobacterium tuberculosis* H37Rv to assess the impact of *Ss* co-infection in individuals with tuberculosis infection (TBI). In contrast, *Ss* infected individuals exhibited elevated levels of type 2 and regulatory cytokines, median concentrations were IL-4 (5.1 pg/mL Vs 7.1 pg/mL), IL-5 (21.2 pg/mL Vs 26.4 pg/mL), IL-10 (142.9 pg/mL Vs 204.3 pg/mL), and IL-13 (47.9 pg/mL Vs 50.6 pg/mL), following *M. tuberculosis* stimulation (**Figure 4**). These findings are consistent with a *Ss* induced immunological milieu that favors Th2 and regulatory responses, potentially at the cost of effective antimycobacterial immunity.

### 3.6 Principal component analysis of cytokine and MGIA responses

**Figure 5:**
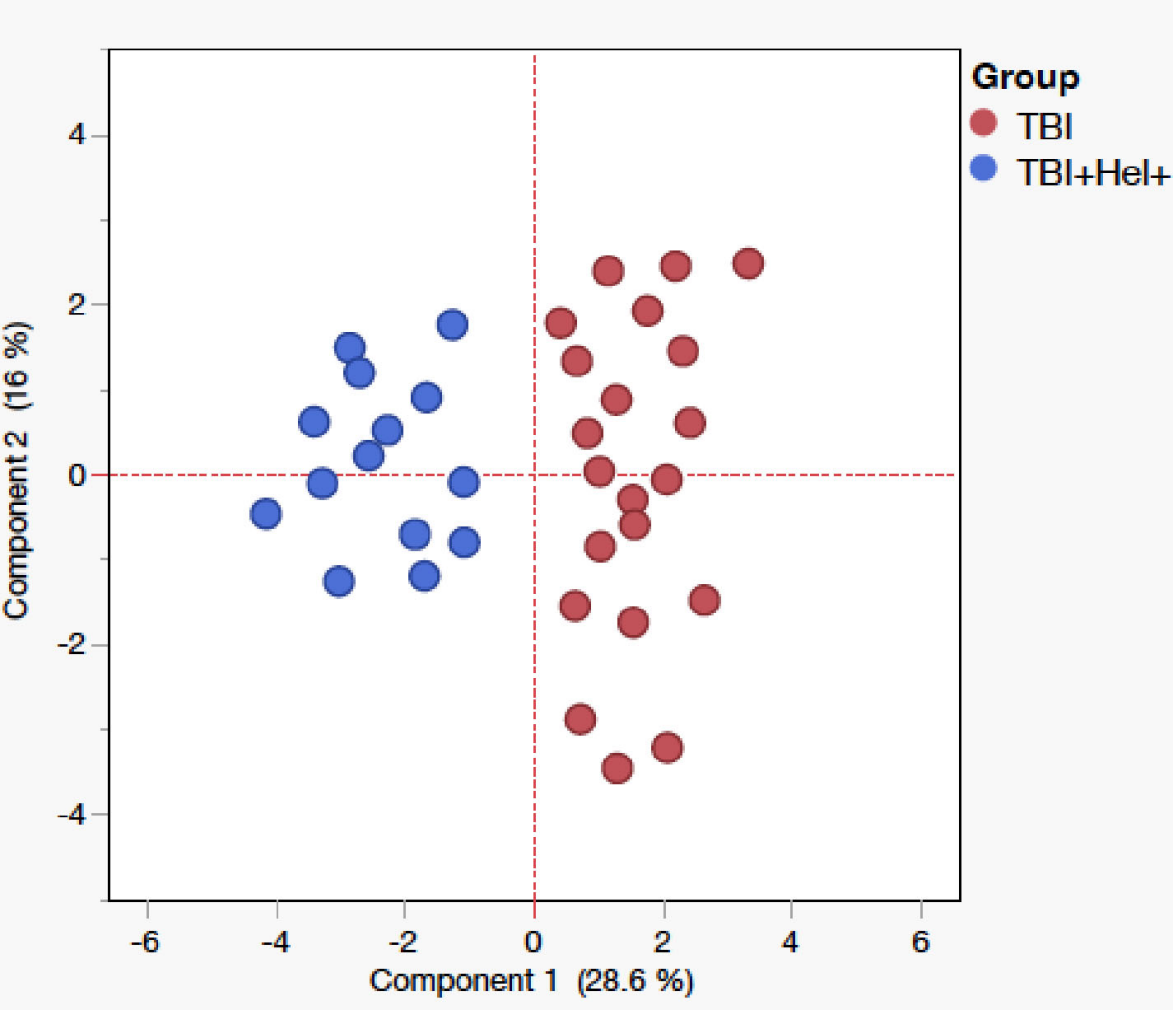
Principal component analysis (PCA) of cytokine and MGIA responses in tuberculosis-infected individuals with or without *Ss* co-infection. Principal component analysis (PCA) was performed using cytokine concentrations and Mycobacterial Growth Inhibition Assay (MGIA) values from tuberculosis-infected (TBI) individuals with helminth co-infection (TBI^+^+Hel^+^) and without (TBI). The first two principal components (PC1: 28.6%; PC2: 16%) together explained 44.6% of the total variance. The PCA biplot shows distinct clustering of TBI and TBI^+^+Hel^+^ groups, indicating helminth-induced immune divergence. Cytokines associated with Th2/regulatory responses (IL-4, IL-5, IL-10, IL-13) clustered positively along PC1, aligning with MGIA values, while Th1/Th17-related cytokines (IL-17, IFN-γ, IL-2, TNF-α, IL-1α, IL-1β) loaded negatively. The segregation suggests that helminth infection reprograms cytokine networks from Th1/Th17-dominant toward Th2/regulatory-dominant profiles, consistent with reduced mycobacterial growth control.

Principal component analysis (PCA) was performed to identify patterns of immune variation associated with helminth coinfection in TBI individuals (**Figure 5**). The first two components (PC1 and PC2) accounted for approximately 44.6% of the total variance (PC1: 28.6%, PC2: 16%), capturing the major immunological differences between the study groups. The PCA biplot showed distinct clustering between TBI and TBI^+^+Hel^+^ group, indicating that *Ss* infection significantly altered the cytokine response network. Cytokines such as IL-4, IL-5, IL-10, and IL-13 loaded strongly on the positive axis of PC1, aligning with MGIA, and reflected a Th2/regulatory-dominant profile in helminth-infected individuals. In contrast, IL-17, IFN-γ, IL-2, TNF-α, IL-1α, and IL-1β clustered negatively along PC1, representing Th1/Th17-associated inflammation characteristic of helminth-negative TBI cases. This segregation highlights that helminth infection promotes a systemic shift from Th1/Th17 to Th2/regulatory dominance, consistent with reduced mycobacterial control.

### 3.7 Relationship between the levels of MGIA and cytokines

Correlation analysis between cytokine profiles and Mycobacterial Growth Inhibition Assay (MGIA) responses in tuberculosis-infected (TBI) individuals with *Ss* co-infection (TBI^+^Hel^+^) revealed distinct immunological associations. As shown in **Table 2** 3II, among the measured cytokines, IL-17 (r = –0.3884, p = 0.0110), IL-1α (r = –0.3537, p = 0.0371), and IL-1β (r = –0.3950, p = 0.0189) displayed significant negative correlations with MGIA, suggesting that elevated levels of these proinflammatory mediators were associated with reduced mycobacterial growth control. Other Th1 cytokines such as IFN-γ, IL-2, and TNF-α showed negative but non-significant trends with MGIA, while Th2 and regulatory cytokines (IL-4, IL-5, IL-10, IL-13) showed weak positive correlations, none reaching significance. Although some correlations reached statistical significance, the Spearman rho values were low, indicating that these associations are relatively weak and should be interpreted with caution. To further evaluate the independent contributions of cytokines and Ss-specific IgG to mycobacterial growth inhibition, all measured cytokines along with *Ss*-IgG were included in a multivariate regression model (**Table 3**). The model demonstrated that *Ss*-IgG, IL-17, IL-1α, and IL-1β remained negatively associated with MGIA values, while selected Type 2 cytokines such as IL-5, IL-10, and IL-13 showed positive associations. These results indicate that despite the presence of inflammatory mediators, the functional ability to restrict mycobacterial growth is impaired in *Ss* -TBI co-infected individuals, pointing toward a qualitative imbalance rather than quantitative deficiency in cytokine responses.

**Table 2:** Relationship between the levels of MGIA and cytokines.

Variables	Spearman’s p	p-value
IFNγ Vs MGIA	-0.1115	0.5231
IL-2 Vs MGIA	-0.2908	0.0901
TNFa Vs MGIA	-0.1252	0.4738
IL-17 Vs MGIA	-0.3884	0.0110
GM-CSF Vs MGIA	-0.1350	0.4394
IL-1α Vs MGIA	-0.3537	0.0371
IL-1β Vs MGIA	-0.3950	0.0189
IL-6 Vs MGIA	-0.0967	0.5803
Il-12 Vs MGIA	-0.2039	0.2401
IL-18 Vs MGIA	-0.1449	0.4061
IL-4 Vs MGIA	0.1504	0.3883
IL-5 Vs MGIA	0.1228	0.4824
IL-10 Vs MGIA	0.3047	0.0751
IL-13 Vs MGIA	0.1178	0.5002

**Table 2** Spearman’s correlation between cytokine concentrations and mycobacterial growth inhibition assay (MGIA) responses in *Ss* –TBI co-infection.

Spearman’s rank correlation coefficients (r) were calculated to assess associations between plasma cytokine levels and MGIA responses in Tuberculosis-infected (TBI) individuals with *Ss* co-infection (TBI^+^+Hel^+^). Significant negative correlations were observed for IL-17 (r = –0.3884, p = 0.0110), IL-1α (r = –0.3537, p = 0.0371), and IL-1β (r = –0.3950, p = 0.0189), indicating that elevated inflammatory cytokines correspond to reduced mycobacterial control. Other proinflammatory cytokines (IFN-γ, IL-2, TNF-α) showed non-significant negative trends, while Th2/regulatory cytokines (IL-4, IL-5, IL-10, IL-13) demonstrated weak positive correlations. These patterns suggest that *Ss* co-infection alters immune functionality, shifting from protective Th1/Th17-driven responses to a Th2/regulatory-biased phenotype.

**Table 3:** Multivariate regression model including *Ss*-IgG values.

Predictor variable	β Coefficient	95% CI	p-value
Ss-specific IgG (OD)	+0.28	0.12 – 0.44	0.001
IFNγ	-0.10	-0.30 – 0.05	0.18
IL-2	-0.14	-0.33 – 0.02	0.09
TNFa	-0.12	0.29 – 0.04	0.13
IL-17	-0.31	-0.52 – -0.10	0.004
IL-22	-0.08	-0.24 – 0.05	0.22
GM-CSF	-0.15	-0.36 – 0.01	0.07
IL-1α	-0.22	-0.40 – -0.03	0.025
IL-1β	-0.29	-0.49 – -0.08	0.007
IL-6	-0.11	-0.29 – 0.03	0.14
Il-12	-0.09	-0.30 – 0.04	0.19
IL-18	-0.05	-0.20 – 0.09	0.48
IL-4	+0.12	-0.01 – 0.28	0.08
IL-5	+0.15	0.02 – 0.32	0.03
IL-10	+0.18	0.04 – 0.33	0.015
IL-13	+0.16	0.01 – 0.29	0.04

Regression coefficients represent estimated direction and magnitude of associations between cytokine levels and MGIA (log10 CFU). Ss-IgG values modelled as continuous OD units.

## 4 Discussion

Our study demonstrates that helminth co-infection significantly compromises protective immune responses in individuals with tuberculosis infection (TBI). Our findings describe immune modulation associated specifically with *Strongyloides stercoralis* co-infection, as this was the only helminth species detected in the study population. Using a mycobacterial growth inhibition assay (MGIA), we observed impaired control of *Mycobacterium tuberculosis* (*M.tb*) H37Rv *in vitro* among TBI- *Ss* co-infected individuals compared to TBI+ individuals. This functional deficiency coincided with a shift in cytokine responses, characterized by reduced Th1, Th17, and innate proinflammatory cytokines, and enhanced Th2 and regulatory cytokine production.

Our data reveal that *Ss* co-infection in tuberculosis-infected individuals significantly alters hematological parameters. Although age, gender, and BMI were comparable between groups, TBI+ Hel+ individuals showed reduced hemoglobin and RBC levels, consistent with helminth-induced anemia due to chronic blood loss and iron deficiency (15). The decrease in lymphocyte counts may reflect helminth-driven immune modulation, where a shift from Th1 to Th2 responses suppresses cell-mediated immunity essential for controlling *Mycobacterium tuberculosis* (16).

Conversely, eosinophil levels were significantly elevated in TBI+ Hel+ individuals, a hallmark of helminth infections. Eosinophilia is driven by IL-5 and other Th2 cytokines, which promote eosinophil proliferation and survival (17). Additionally, helminth-induced Th2 polarization can dampen protective Th1 responses against TB, potentially impairing host defense mechanisms (18). These data emphasize the importance of considering helminth co-infection in TB-endemic regions, as it may influence disease progression, immune response, and treatment outcomes.

Our data demonstrates that in individuals with tuberculosis infection (TBI), coexisting *Strongyloides stercoralis* infection has been associated with a significant decrease in antigen-specific type 1, type 17, and pro-inflammatory cytokine responses. While mitogen-induced responses remained unchanged. As expected, PHA stimulation did not reproduce the differences observed with TB antigen stimulation, likely because mitogenic activation bypasses antigen-specific pathways that are more susceptible to helminth-associated modulation. No additional antigenic stimuli were evaluated beyond QFT TB1/TB2 peptides and H37Rv lysate. Cytokine responses, while reduced overall in the *Ss* infected group, varied depending on whether stimulation was performed with QFT antigens or H37Rv.

The stronger group differences observed with TBAg2 likely reflect the broader CD4^+^ and CD8^+^ T-cell stimulation captured by the TB2 tube, which may be more sensitive to *Ss* associated immune suppression, including effects on IL-1 family cytokines. Decreased IL-1α and IL-1β indicate weakened innate inflammatory signaling, while decreased Th1 and Th17 cytokines emphasize compromised macrophage activation and early mucosal defense. Th1 cytokines such as IFN-γ, IL-2, and TNF-α are critical for macrophage activation and granuloma maintenance, playing indispensable roles in controlling *M.tb* replication and dissemination (19) (20) (21). The observed reduction in these cytokines in helminth-infected individuals aligns with earlier reports suggesting helminth-driven suppression of Th1 responses (22, 23). Additionally, IL-17 and GM-CSF, both important for neutrophil recruitment and epithelial defense (24, 25), were significantly lower in helminth-co-infected individuals, suggesting impaired mucosal barrier integrity and granuloma function. Our findings align with those of Anwar et al. (26), who also reported reduced mycobacterial growth inhibition in individuals with predominantly *Strongyloides stercoralis* infection. In contrast, Shea et al. (27) observed improved MGIA in individuals with hookworm infection, which reverted to control levels following anthelmintic treatment. These contrasting results suggest that different helminth species may exert distinct effects on antimycobacterial immunity, and that helminth–TB interactions are not uniform across infections.

Innate cytokines such as IL-1α, IL-1β, IL-6, IL-12p70, and IL-18 were also significantly reduced. These cytokines initiate and amplify early immune responses and are essential for bridging innate and adaptive immunity (28, 29). Suppression of these mediators by helminths may delay or dampen the immune priming necessary for robust anti-TB responses. In contrast, we observed elevated IL-4, IL-5, IL-10, and IL-13 in the helminth-infected group, consistent with a Th2/regulatory bias. Such cytokines promote tissue repair and immune regulation but can inhibit effective antimicrobial activity (30). Notably, IL-10 has been associated with TB reactivation and persistence due to its suppressive effect on macrophage and T-cell function (31). Recent studies further confirm that helminth infection induces a Th2/regulatory environment, characterized by increased IL-10 and impaired Th1/Th17 responses, which can compromise anti-mycobacterial immunity (5).

Our data extends our earlier finding that *Ss* infection exacerbates tuberculosis infection (TBI) by revealing that helminth co-infection is associated with both qualitative alterations in cytokine–MGIA correlations and distinct immunological clustering by PCA. The significant negative associations between IL-17, IL-1α, and IL-1β and MGIA responses suggest that heightened inflammatory signaling is not protective but instead reflects immune dysregulation, leading to reduced bacterial control. Helminth infections are well known to induce Th2 and regulatory immune polarization, characterized by increased IL-4, IL-5, IL-10, and IL-13 production and expansion of T regulatory cells, which suppress Th1/Th17 effector responses critical for antimycobacterial immunity (23, 32, 36). The PCA-supported shift toward a Th2/regulatory cytokine signature further indicates that helminth co-infection reprograms immune homeostasis, promoting a milieu that favors chronic tolerance rather than pathogen clearance. Although IL-10 and IL-13 showed weak positive associations with MGIA, these likely represent compensatory anti-inflammatory feedback mechanisms that inadvertently dampen effective Th1-mediated protection. Collectively, these findings demonstrate that *Ss* infection skews host immunity away from protective Th1/Th17 balance, resulting in impaired functional control of *Mycobacterium tuberculosis*. This immune reprogramming may partly explain reduced vaccine responsiveness and poor TB outcomes in helminth-endemic settings (33–35). Multivariate model confirmed that *Ss* driven biomarkers such as *Ss* IgG, IL-17, IL-1α, and IL-1β contributed independently to impaired mycobacterial growth control, while elevated Type 2 cytokines (IL-5, IL-10, IL-13) showed positive associations with MGIA, further supporting a *Ss* associated shift away from protective Th1/Th17 pathways.

Our study has certain limitations. The sample size is relatively small. Furthermore, a causal relationship between *Ss* co-infection and compromised *Mycobacterium tuberculosis*-specific immunity cannot be inferred due to the cross-sectional design. Participants were not categorized by helminth species, burden, infection severity, or treatment history—all of which are known to affect immune modulation. TBI controls were not included because the study focused on TBI cases, and TBI–*Ss*+ individuals were rare in this population. Only the mycobacterial growth inhibition assay was used, without the use of complementary cellular assays like T-cell phenotyping or intracellular cytokine staining, and cytokine responses were evaluated at a single time point, which might not adequately capture the dynamic nature of immune responses.

Our study shows that shifting responses from proinflammatory Th1/Th17 to Th2/regulatory pathways, helminth co-infection reduces TB-specific immunity in TBI patients. These immune alterations may influence the performance of TB diagnostics and vaccines in helminth-endemic areas. Our findings emphasize the necessity of taking helminth burden into consideration when developing TB control strategies. They also highlight the significance of integrated intervention approaches and the need for further research to elucidate the mechanistic pathways that connect helminth infection, immune modulation, and TB risk.

## Data Availability

The original contributions presented in the study are included in the article/supplementary material. Further inquiries can be directed to the corresponding author.
